# Prioritizing Environmental Issues around the World: Opinions from an International Central and Eastern European Environmental Health Conference

**DOI:** 10.1289/ehp.9300

**Published:** 2006-08-18

**Authors:** Elena S. Craft, Kirby C. Donnelly, Iulia Neamtiu, Kathleen M. McCarty, Erica Bruce, Irina Surkova, David Kim, Iveta Uhnakova, Erika Gyorffy, Eva Tesarova, Beth Anderson

**Affiliations:** 1 Nicholas School of the Environment, Duke University, Durham, North Carolina, USA; 2 Laboratory of Molecular Toxicology, National Institute of Environmental Health Sciences, National Institutes of Health, Department of Health and Human Services, Research Triangle Park, North Carolina, USA; 3 Department of Veterinary Anatomy and Public Health, Texas A&M University, College Station, Texas, USA; 4 Environmental Health Center, Cluj-Napoca, Cluj County, Romania; 5 Department of Epidemiology, University of North Carolina at Chapel Hill, Chapel Hill, North Carolina, USA; 6 Department of Civil Engineering, Environmental and Water Resources Division, Texas A&M University, College Station, Texas, USA; 7 Department of Medical Genetics, Sechenov Moscow Medical Academy, Moscow, Russia; 8 Department of Environmental Sciences and Engineering, School of Public Health, University of North Carolina at Chapel Hill, Chapel Hill, North Carolina, USA; 9 Department of Environmental Medicine, Research Base of the Slovak Medical University, Bratislava, Slovakia; 10 National Institute of Environmental Health, Fodor Jozsef National Center for Public Health, Budapest, Hungary; 11 Department of Physical and Macromolecular Chemistry Faculty of Science, Charles University, Prague, Czech Republic; 12 National Institute of Environmental Health Sciences, National Institutes of Health, Department of Health and Human Services, Research Triangle Park, North Carolina, USA

**Keywords:** Eastern Europe, environmental health, environmental pollutants, international health concerns

## Abstract

**Background:**

As the next generation of scientists enters the field of environmental health, it is imperative that they view their contributions in the context of global environmental stewardship. In this commentary, a group of international graduate students facilitated by three experienced environmental health scientists present their views on what they consider to be the global environmental health concerns of today. This group convened initially in October 2004 at an international health conference in Prague, Czech Republic.

**Objectives:**

In this report we identify perceived environmental health concerns that exist around the world, with a focus on Central and Eastern Europe. Additionally, we address these perceived problems and offers some potential solutions.

**Discussion:**

At the meeting, students were invited to participate in two panel discussions. One group of young international scientists identified several significant global environmental health concerns, including air pollution, occupational hazards, and risk factors that may exacerbate current environmental health issues. The second panel determined that communication, education, and regulation were the mechanisms for addressing current environmental challenges.

**Conclusions:**

In this commentary we expand on the views presented at the meeting and represent the concerns of young investigators from nine different countries. We provide ideas about and support the exchange of information between developed and developing countries on how to handle the environmental health challenges that face the world today.

There is an increasing need to prevent or mitigate known exposures, extrapolate environmental remediation knowledge, and share lessons of effective risk communication from industrialized countries with less developed areas of the world. To facilitate this process, global health concerns resulting from environmental exposures need to be addressed universally. Additionally, the global scientific community must commit to working together to help find solutions to these health issues as they are experienced throughout the world. If the next generation of health scientists is to act as stewards of the environment, this group must place more emphasis on sharing existing knowledge with all nations, specifically by collaborating with other nations that are dealing with the same environmental challenges.

The International Central and Eastern European Health Conference was held in Prague, Czech Republic, in October 2004. This location was selected because current social, political, and economic transitions in this region have resulted in a dramatic impact on environmental and occupational exposures. During the past two decades, the period of political change, inadequate/aging infrastructure, and a lack of economic resources in Eastern and Central Europe have resulted in increased pollution and public health risk (Bell 2000; Organization for Economic Cooperation and Development 1999). A lack of regulation of environmental pollution and an increase in poor industrial and agricultural practices have resulted in increased air and water pollution as well as soil contamination (Jedrychowski 1995; Woolfson 2006).

The purpose of this commentary is to summarize some of the main conference themes that were identified by the international graduate students who participated in the platform discussions at the conference. These students were asked to share their opinions and to prioritize the predominant environmental issues facing their respective countries, and to provide potential solutions for them. These forums identified societal and political issues as a potential challenge to implementing environmental reforms, whereas education and training were described as some of the most immediate ways to initiate action on these issues. Additionally, these two groups also emphasized the prominent need to address children’s health issues, because children are often considered the most susceptible subgroup of any population. We hope that this commentary will highlight the international nature of current environmental health problems and underscore the vital need to increase the exchange of information across borders and scientific disciplines.

## Panel 1: Identification of Environmental Health Problems

As a diverse group, we readily identified numerous environmental factors that contribute to disease, dysfunction, or poor health. Although our individual perspectives vary, the two panels were unanimous and consistent in identifying the same group of environmental problems. We divide our discussion of these problems into four parts: *a*) air pollution specifically as it exacerbates respiratory function and morbidity from asthma and other conditions; *b*) occupational hazards; *c*) poor environmental conditions produced by the release of metals from mining and industrial activities, contamination of drinking water by chemicals and infectious agents, and the release of endocrine disruptors and pesticides into the environment; and *d*) other health issues that are exacerbated by certain lifestyle factors, including drinking, smoking, and unhealthy diet.

### Air pollution that exacerbates cardiopulmonary and respiratory diseases

High-concentration, short-term exposure to air pollutants (particulates, nitrogen dioxide, and sulfur dioxide) may increase mortality in the population, particularly among those that are susceptible to these effects. Susceptible subpopulations include individuals with chronic obstructive pulmonary diseases (COPD) such as ischemic heart diseases, congestive heart failure, heart rhythm disorders, asthma, and diabetes (Lagorio et al. 2006). In Cracow, Poland, it was estimated that an increase of SO_2_ concentrations of 100 μg/m^3^ was associated with a 19% increase in daily mortality related to respiratory system diseases, whereas deaths due to circulatory system disease increased by 10% (Jedrychowski 1995). Studies conducted in the Czech Republic and Poland show that air pollution is associated with increased overall mortality, postneonatal mortality, and lung cancer (Bobak and Marmot 1996).

**Figure 1:**
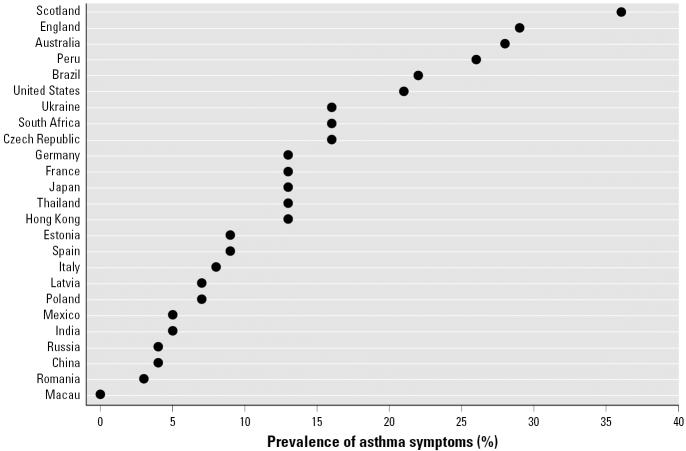
Prevalence of asthma symptoms in childhood by country (written questionnaire: self-reported wheezing in the previous 12-month period, in 13- to 14-year-old children). Adapted with permission from Global Burden of Asthma Summary (2002).

Air pollution is known to exacerbate many respiratory conditions. Asthma, for instance, is the most common chronic childhood disease in many countries and it affects children and adults worldwide. The increase in asthma in English-speaking countries and the international pattern of the disease suggests the role of environmental factors in the etiology of asthma. In 1998, the International Study of Asthma and Allergies in Childhood (ISAAC) Steering Committee estimated the prevalence of childhood asthma as 17–30% in the United Kingdom, New Zealand, and Australia, compared with 1–7% in Eastern Europe, China, and Indonesia (ISAAC 1998). More recent data from the Global Initiative for Asthma demonstrates a great degree of variability in the prevalence of childhood asthma across global populations (Figure 1) (Global Burden of Asthma Summary 2002), while identifying substantial increases in some Eastern European nations.

Worldwide, asthma accounts for 1 in every 250 deaths. This increase in cases of asthma has been partially attributed to a complex set of environmental factors associated with urbanization, including xenobiotic exposures (Global Burden of Asthma Study 2002). Additionally, other health effects such as obesity or poor diet (Black and Sharp 1997), childhood respiratory diseases (Lemanske and Busse 2006), genetics (Arruda et al. 2005), and emotional stress (Bloomberg and Chen 2005) may influence the severity of an asthmatic condition. Because the prevalence of asthma is increasing and environmental influences are known to exacerbate this disease, it is imperative that a multinational approach be taken to address the rising health care challenges associated with treating this disease.

### Occupational hazards

Occupational exposure limits and safety protocols are standard in most industrialized countries. However, many developing countries lack standards for worker protection. Workplace exposure to dust, metals, silica, gases such as SO_2_ and NO_2_, and other hazardous substances continues to be a major health and safety problem in Central and Eastern Europe. These standards appear to be effective as developed countries generally record lower frequencies of workplace-related diseases, such as lead poisoning, asbestosis, COPD, and pneumoconiosis [World Health Organization (WHO) 2001].

In 1996, the World Health Assembly adopted the Global Strategy on Occupational Health for All, which identified major work-force hazards (WHO 1995). Several environmental hazards, such as biologic agents, reproductive hazards, occupational carcinogens (e.g., asbestos), and allergenic agents were included in the list. The International Labor Organization and the WHO reported that occupational accidents account for 333,000 fatalities worldwide per year (WHO 1995). Many of these fatalities are a result of exposure(s) to chemical (e.g., benzene, chromium), physical (e.g., ultraviolet radiation, ionizing radiation), or biologic (e.g., aflatoxin, tumor viruses) hazards that may be present in the work environment (WHO 1995).

### Environmental pollutants

#### Metals

Of the natural or anthropogenic metals that are released into the environment, lead is of great concern, especially with regard to exposures in children. Ingestion or inhalation of lead by small children may damage cognition and behavior and can cause developmental delay and mental retardation (Jarosinska et al. 2006; Valent et al. 2004). In polluted areas of Slovakia (near metallurgic and glass factories), for instance, children’s blood lead levels were found to be dramatically higher than in non-polluted areas. In addition, children living near a metallurgic plant performed poorly on intelligence tests compared with children from non-polluted areas (Sovcikova and Wsolova 2000).

Other sources of metal exposures include drinking water, indoor burning of coal, and ingestion of contaminated foods. For example, millions of people are exposed to arsenic globally, which is associated with many detrimental health effects such as several types of cancer, diabetes mellitus, and vascular, reproductive, developmental, and neurologic effects (National Research Council 2001).

#### Contaminated drinking water

Infectious diseases associated with drinking contaminated water is a problem in many parts of the world. It is estimated that poor water quality, sanitation, and hygiene result in 1.7 million deaths per year internationally (Ashbolt 2004). Microbial contamination of drinking water also remains a concern in several regions of Europe. In Central and Eastern Europe and Western Asia, it is estimated that > 5% of all childhood deaths are attributable to diarrheal disease, which is often a result of poor-quality drinking water, inadequate sanitation, or improper personal hygiene (Valent et al. 2004).

Although there is concern over the microbial contamination in drinking water in some areas, the presence of metals in drinking water is also a significant health threat. Contaminants in surface and groundwater may come from anthropogenic sources, runoff from agricultural activities (Chapin et al. 2005), or controlled or uncontrolled discharges from sewage treatment facilities or leaking landfill sites (Kolpin et al. 2002). Arsenic contamination of groundwater is a problem in many parts of the world. Specific to Central and Eastern Europe, elevated levels of arsenic in drinking water have been detected in Slovakia, Hungary, and Romania (Lindberg et al. 2006).

### Endocrine-disrupting compounds (EDCs) and pesticides

In recent years, it has become apparent that the environmental presence of chemicals that mimic natural hormones can have deleterious effects on reproduction in ecologic species. A broad range of synthetic compounds including some plasticizers and organochlorine pesticides have been found to have estrogenic activity. Although EDCs clearly affect wildlife populations, conflicting results have been observed in human studies comparing tissue levels of these compounds with sperm viability or the incidence of breast cancer. Chronic exposure to phytoestrogens and xenoestrogens may produce acceleration of pubescence, increased incidence of vaginal and prostate cancers in adults, and alterations in sexually dimorphic anatomy, physiology, and behavior (Safe 2005).

The agricultural use of organochlorine pesticides has been banned in most countries. However, because of public health needs, these compounds are still in use in many regions to control vectors of infectious disease. Thus, although many developed countries are currently trying to assess the chronic health impact of residual organochlorine pesticides on human health, developing countries are still using organochlorines to prevent acute infectious disease. Levy and Wegman (2000) indicate that the potential effects associated with exposure to organochlorine pesticides include neurobehavioral disorders, cancer, and spontaneous abortions. Often, populations in developing countries are in more intimate contact with their environment, which may result in an increased risk of exposure, leading to adverse health effects.

### Risk factors and environmental health problems

In addition to the environmental health concerns mentioned, we identified several other risk factors that can exacerbate existing environmental health issues. Alcoholism, smoking, and obesity were discussed specifically as lifestyle choices that may increase the severity of existing medical conditions caused by poor environmental health.

We were especially concerned with the increase in alcoholism among young adults. According to a WHO report (2004), the European Region has the highest alcohol intake in the world. In this region, alcohol abuse accounts for one of four deaths among men, and one of 10 deaths among women, in the population 15–29 years of age (WHO 2004). Nemtsov (2005) estimated that up to 30% of deaths in Russia can be directly or indirectly attributed to alcohol. Adolescents may be particularly susceptible to the influences of alcohol due to social pressures. In a comprehensive study of family structure and alcohol use among 15- to 16-year-old students in 11 European countries, for instance, Bjarnason et al. (2003) found that adolescent heavy drinking is more common in broken homes or disrupted families.

Additionally, although the use of tobacco has declined in many industrialized countries, its use is increasing in developing countries. The WHO estimates that by 2020, the total number of deaths attributable to tobacco around the world will double due to the increase in tobacco use in former socialist economies and demographically developing countries (Table 1) (WHO 1996). Historically, these societies have been low- or middle-income economies and presumably less well informed on the risks of tobacco use. Smoking among adolescents is also of great concern. An extensive study on Hungarian, Polish, Turkish, and American adolescents by Piko et al. (2005) shows that complex interrelationships between adolescents’ smoking and its social and personal influences are similar across all countries, regardless of culture or smoking rates.

**Figure 2:**
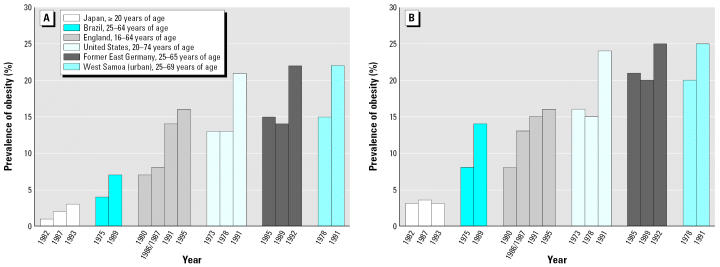
Prevalence of obesity in adults: (*A*) men; (*B*) women.

Obesity was a third lifestyle factor presented as a serious threat to the health of those already affected by poor environmental conditions. Reports from the Centers for Disease Control and Prevention in the United States indicate that the incidence of obesity, as defined by a body mass index of ≥30, has more than doubled in the United States from slightly > 13% in 1960–1962 to > 31% in 1999–2002 (National Center for Health Statistics 2004). Similar numbers were reported for other countries around the world (Wilding 1997). Figure 2 demonstrates this growing epidemic and the prevalence worldwide (International Union of Nutritional Sciences 2002). Environmental exposure may be a co-factor in determining disease outcomes related to obesity because it has the potential to exacerbate medical conditions, such as diabetes and cardiovascular disease, in addition to having other deleterious health effects. Recent evidence suggests that obesity predisposes individuals to developing asthma and other illnesses, including some types of cancer, stroke, kidney and gallbladder disease, and osteoarthritis (Burton et al. 1985), all of which are considered to have possible environmental components.

Our panel came to the consensus that the health effects resulting from poor environmental conditions are greatly exacerbated by risk factors that include alcohol, smoking, and obesity.

## Panel 2: Potential Solutions to Environmental Health Problems

The second panel focused on discussions regarding appropriate and viable resolutions to the perceived international environmental health challenges. We recognized many difficulties in changing social, cultural, and political structures to promote environmental awareness and ecologic health; however, it was evident that research alone is not sufficient to address these issues. Our discussion focused on communication, education, and regulation as the key factors for improving human health and the environment.

### Communication

Environmental health scientists must improve the translation of research results to the public. We believe that one of the major responsibilities of scientists to society is to communicate their results accurately, effectively, and in a timely manner both to the public and to policy makers. Many individuals within the general population may lack the education or expertise needed to interpret environmental health research. In addition, it is not uncommon for different scientists to interpret the same data quite differently. Thus, it is important when communicating with the public that scientists present a balanced summary of the available information, including conflicting opinions when necessary. Collaboration with the media can be an effective resource for increasing public awareness. Furthermore, the media may serve as a mechanism to improve the understanding among policy makers with regard to environmental health issues.

One example of such a media-based program was conducted in Russia, Hungary, the Czech Republic, and Poland between 1996 and 1998. A five-part television series was developed by local health professionals and focused on the promotion of a healthy diet, enhanced physical activity, and avoidance of tobacco and alcohol. Subsequently, 16.7–45.0% reported actual dietary changes consistent with health promotion (Chew and Palmer 2005). These results suggest that television programs on health promotion are a tool for increasing public awareness in many countries including those in Central and Eastern Europe.

Effectively communicating global health risks to the public is a significant challenge. A recent study conducted in several Eastern European countries examined the differences of the perception of risk with respect to environmental issues. Slachtova et al. (2003) investigated how two adjacent countries differed in their views of environmental health priorities. According to their study, citizens in one of the countries expected the solutions to environmental health issues to be addressed by the government, whereas citizens of an adjacent country believed that citizens should play a more active role in responding to environmental problems. Additionally, those in the first country tended to focus on the perception of local problems, whereas those in the second considered environmental and health problems on a wider national, and even global, scale. This study also identified nongovernmental organizations and local scientists as the most credible sources of information in both countries. This suggested that these institutions could play an important role in risk communication.

Risk perception is a complicated issue, and each society will select approaches for managing risk according to its own values and priorities. However, the harmonization of approaches, where freely accessible information is exchanged among international experts, will permit choices to be made based on the most reliable information available and will encourage national environmental health concerns to be addressed as a global responsibility rather than isolated environmental insults.

### Education

Education is paramount in the prevention of disease as related to environmental health. A broad definition of environmental health encompasses not only the adverse effects associated with exposure to chemicals in air, food, and water, but also the interaction of these exposures with genetic sensitivities and nutrition. Although environmental education is emerging on all educational levels, including elementary schools, efforts need to be expanded, with emphasis on addressing the multidisciplinary nature of environmental rehabilitation in the context of disease prevention. For instance, experts in environmental science should participate in classroom lectures as well as workshops for teachers. These lectures could range from educational programs for children and young adults as part of a science curriculum to graduate-level education in environmental health.

Furthermore, we identified a need to train health professionals in recognizing that many health problems have an environmental component. This need exists in both developed and developing countries. Additionally, training both management and workers on how to mitigate environmental exposures in the work-place must be a priority. Whenever possible, it would be useful to obtain data from biologic and environmental monitoring to evaluate the efficacy of educational efforts in reducing occupational exposures.

Appealing to the public via social influences and businesses that promote sustainable development, for instance, could be another way to present information on environmental health concerns. Providing more environmentally responsible alternatives, such as incorporation of sustainable development practices, may help increase awareness on some of the global environmental health concerns that have been mentioned here. For sustainable development to work, however, effective protection of the environment, practical use of resources, maintenance of high and stable economic growth and employment, and protection of public health and social impacts must be considered (Sachs and Reid 2006).

### Regulation

Regulation is a more complicated solution to addressing environmental problems, but is critical in attaining the desired outcomes. Although regulation is important, many obstacles exist in setting environmental policies. Establishing a regulation requires a governing body that is adequately informed of the importance of environmental issues and the need for policy directives. Once in place, the regulations require an infrastructure that supports implementation. Adhering to environmental regulations may be difficult if not impossible for countries with unstable governments or countries that do not have the resources to support such organization and maintenance of regulations. The existence of an agency to ensure compliance is another challenge in enforcing environmental legislation. Other social or political issues such as economic factors and political agendas are potential obstacles to solving difficult environmental problems or promoting healthy lifestyle changes.

Despite the economic cost and infrastructure needed to create and maintain environmental regulations, they are an essential part of protecting populations from environmental health hazards. New studies are regularly being conducted to investigate the complex relationships between health and the environment. Accordingly, it is important that scientists ensure that the most current and accurate information is readily available to policy makers at both the national and international level.

## Conclusion

Due to the increasingly multinational nature of environmental insults, we as young scientists needed to address these issues at the international level by building an infrastructure to prioritize environmental problems. Here, we acknowledge the challenges that face the world as a whole, including the difficulties in sharing information freely, the difficulties in crossing cultural and language barriers, and the difficulties in maintaining sensitivity to the concerns of each nation. Political agendas and constraints on financial resources routinely lead to disagreements in the prioritization of environmental problems and may interfere with creating needed legislation. We must learn how to negotiate these circumstances to achieve a viable level of environmental protection both regionally and globally.

It is the responsibility of all citizens to overcome these barriers and create a more unified approach to addressing global problems. It is the responsibility of scientists to accurately characterize environmental health problems and to identify appropriate ways to solve these problems. Computers, analytical instrumentation, microarray technologies, and molecular technology provide scientists with tools to look inside a cell and identify mechanisms of disease. It is the responsibility of young scientists to use interdisciplinary research and these advanced technologies to bring together innovative approaches to solving issues such as predicting risk and reducing exposure. These innovative approaches will play a crucial role in addressing challenges posed by environmental contamination. We also believe that basic research will not resolve environmental problems in isolation, but must be pursued with awareness of societal needs and in the context in which these issues exist.

Additionally, we must recognize that developing nations themselves have expertise to impart in the quest to fulfill our scientific responsibilities, and that we must change environmental habits by sharing knowledge and resources. It should be a goal of all environmental health scientists to respect the political and economic needs of countries in the initial stages of expanding their industrial resources. Communication among scientists is also important to prevent developing countries from repeating the same mistakes that have been made in nations that have a more established industrialized base. We hope the dialogue presented here indicates our commitment to relay the perceived health concerns and potential solutions to other scientists and environmental policy makers around the world.

## Figures and Tables

**Table 1:** Econometric model projections of deaths and disease burden attributable to tobacco, estimates for 1990 and projections for 2020.

	Total deaths (thousands)		Deaths (percent of total)	
Region	1990	2020	1990	2020
Established market	1,063	1,286	15	15
Former socialist economies	515	1,101	14	23
Demographically developing countries	1,460	5,996	4	11
World	3,038	8,383	6	12
